# Value of the Brain and Spinal Injury Center Score in Assessment and Prognosis of Acute Traumatic Spinal Cord Injury

**DOI:** 10.1089/neur.2023.0112

**Published:** 2024-07-01

**Authors:** Temitope I. Babalola, Salman A. Yusuf, Mahmud Raji, Jimoh O. Kamaldeen, Duro Dolapo

**Affiliations:** ^1^ Neurosurgery Unit, Nisa Premier Hospital, Jabi, Nigeria.; ^2^ Neurosurgery Unit, National Hospital, Abuja, Nigeria.; ^3^ Radiology Department, National Hospital, Abuja, Nigeria.; ^4^ Department of Community Medicine, Nile University, Abuja, Nigeria.

**Keywords:** AIS, BASIC score, magnetic resonance imaging, spinal cord injury, trauma

## Abstract

The objective was to assess the severity of neurological injury in acute traumatic spinal cord injury (ATSCI) using the BASIC (Brain and Spinal Injury Center) score, to correlate with the American Spinal Injury Association (ASIA) Impairment Scale (AIS) grade at admission and at 3 months postinjury in patients managed for ATSCI at National Hospital, Abuja, and thereby validate the novel BASIC score. This was a prospective longitudinal hospital-based study involving consecutive patients diagnosed with ATSCI and managed at the National Hospital, Abuja. Sixty-five participants met the inclusion criteria. Each patient was resuscitated along the Advanced Trauma Life Support protocol, followed by history, neurological examination according to the International Standards for the Neurological Classification of Spinal Cord Injury (ISNCSCI), and AIS grades that were recorded. Magnetic resonance imaging scan of the injured spinal cord was done, and BASIC scores were assigned. Further management was as per the standard. Three months after injury, neurological examination was again carried out based on ISNCSCI and AIS grades assigned. Data were collected, analyzed, and correlated using Excel and SPSS version 23. Means, medians, correlation coefficients, and Fisher’s exact *t*-tests were determined. *p*-Value <0.05 was considered statistically significant. Results show mean age was 39.1 ± 12.3 years. The majority (81.5%) were males, whereas 18.5% were females. The majority (67.7%) were skilled professionals, 13.8% were unskilled, and 18.5% were students. Most injuries (90.8%) were due to road traffic accidents, whereas 9.2% were due to falls. Majority (72.3%) of the patients had complete SCI (AIS grade A), whereas AIS grade E accounted for the least number (3.1%). Cervical spine injury affected 92.3% of patients, whereas 7.7% had thoracic spine injury. Most patients had BASIC 4 pattern on MRI (44.6%), whereas BASIC 1 pattern was the fewest (3.1%). Surgery was not done for 58.5% of patients, whereas 41.5% had surgical decompression and spine fusion. At 3 months postinjury, 15.4% of patients had AIS grade improvement, whereas 84.6% maintained their AIS grade. The largest AIS grade improvement was from grade B to C (6.2%), which was statistically significant (*p* = 0.04). BASIC score correlated moderately with admission AIS grade (*p* = 0.532). BASIC score also correlated moderately with AIS grade at 3 months postinjury (*p* = 0.546). BASIC score 4 was best at predicting poor outcome in ATSCI. In conclusion, BASIC score has a moderate correlation with AIS grade in ATSCI and can predict poor outcomes in ATSCI. BASIC score of 4 has the best discriminant value in prognosticating and represents severe SCI.

## Introduction

Acute traumatic spinal cord injury (ATSCI) can be defined as a damage to the spinal cord by an insult of a traumatic nature, resulting in transient or permanent loss of usual spinal motor, sensory, and autonomic function.^1^ Spinal cord injury (SCI) is devastating and has functional, psychological, and financial implications for individual patients, families, and society.^2,3^ Shortened life expectancy occurs as a result of multiple complications of the disease.^4^ Direct cost of initial hospitalization in an advanced country such as the United States is estimated at $140,000, whereas indirect costs in the form of lost productivity are up to $2.6 billion annually.^5,6^ An estimate of the cost of conservatively managing a patient with SCI in the acute phase and for a maximum of 6 weeks at a tertiary hospital in north central Nigeria was estimated at $1598.29, representing 6.4–232.8% of average annual income.^7^

SCI research is challenged by unpredictable neurological injury classification that affects the choice of patients randomized for clinical trials. Furthermore, the International Standards for the Neurological Classification of Spinal Cord Injury (ISNCSCI) and American Spinal Injury Association (ASIA) Impairment Scale (AIS) developed and revised by ASIA for patient evaluation perform poorly in predicting recovery after SCI.^8^

With widespread use of patho-anatomical imaging in the form of magnetic resonance imaging (MRI), the exact point(s) of SCI corresponding to functional impairment can be identified. Also, different pathological markers of injury such as edema and hemorrhage show up *in situ* within the damaged spinal cord where they are seen as various signal morphologies, for example, on T2-weighted (T2W) images hyperintensity corresponds to edema, and hypointensity corresponds to hematoma. Identification of these allows rational correlation with clinically determined extent of functional impairment and may allow some prognostication. A particular measure that allows all of these is the BASIC (Brain and Spinal Injury Center) score.^9^ Named for the Brain and Spinal Injury Center of the San Francisco General Hospital, San Francisco, California, USA, it uses MRI T2W imaging features as parameters for injury classification and prognostication of recovery. It is scored as 0, 1, 2, 3, and 4 in increasing order of worsening injury severity. BASIC 0 is when the axial view of the spinal cord on T2W MRI shows normal image of the white and central gray matter; BASIC 1 is when there is edema involving only the full outline of the central gray matter; BASIC 2 is when there is edema only, and it goes beyond the outline of the central gray matter but not involving the full extent of the circumferential white matter; BASIC 3 is when there is edema only, and it involves the full extent of the white matter; BASIC 4 is when there is any hemorrhage within the spinal cord. The finding of intramedullary hemorrhage in addition to BASIC 1, BASIC 2, or BASIC 3 will lead to upscoring by 1.

## Statement of the Problem

Traumatic SCI is a serious cause of morbidity and mortality globally. The AIS, though widely used, even as an outcome measure in clinical trials, has limitations relating to its sensitivity, reliability, and the validity of observations made.^8^ For example, its motor and sensory components were found to be less reliable in classifying incomplete SCI compared with complete SCI.^10–12^ Also, some patients with acute SCI are polytraumatized, unconscious, intoxicated, intubated, or sedated, and up to 80% of patients with traumatic SCI suffer multisystem trauma defined as Abbreviated Injury Score of 3 or more to more than one body region, or an injury severity score of 16 or more,^13^ complicating the clinical examination.

The frequency of head injury in patients with ATSCI is up to 26–74%.^14,15^ According to Bowman B et al.,^14^ 34% of the patients in their prospective series had mild head injury associated with ATSCI, whereas 26% had serious head injury associated with traumatic SCI.

Head injury was especially associated with cervical spine injuries. These particular patients have increased risk of secondary neurological injury from hypoxemia and hypoxia. These clinical states detract from the accuracy, reliability, and outcome of the clinical neurological examination and, by extension, the prognosis. There are no early noninvasive surrogate markers of SCI to assist with diagnosis and injury severity classification in the acute period when there is anxiety for prognosis.

Nigeria has a significant burden of ATSCI.^16^ But to the best of our knowledge, there are a few studies in our environment that have explored the possibility of MRI findings as surrogate markers for injury severity classification and prognostication in ATSCI. There is, therefore, a need for more research in this area.

## Justification

Baseline MRI is recommended as a preoperative investigation necessary for decision-making and outcome prediction in ATSCI,^17^ and this has created a knowledge gap to find features or measures on MRI that will be effective for outcomes’ prediction.

In the acute phase, spinal cord T2 signal changes are associated with various pathological features both in human and in animal studies.^18,19^ T2 signals, although being nonspecific, reflect a combination of vasogenic edema, cytotoxic edema, axonolysis, myelinolysis, inflammatory exudate, and petechial hemorrhage.^19,20^ The much used and evaluated sagittal intensity pattern has been found to be accurate in limited areas of its spectrum,^21^ whereas translational studies in rats have shown strong correspondence between axial T2 signal patterns and functional recovery.^22^ The BASIC score is derived from axial T2 signal patterns.

There is, however, a paucity of studies on the transverse pattern of axial T2 cord MRI signal, especially in our local environment, probably because of the historically low signal-to-noise ratio obtainable with earlier lower strength MRI machines.^23^ In the light of the limitations of previous studies based on sagittal cord signal features,^21^ and the paucity of studies on axial T2 signal patterns, this prospective study attempted to locally validate the novel BASIC score.

## Aims and Objectives

### General objective

The objective of this study was the assessment of severity of neurological injury using the BASIC score and correlation with AIS at admission and 3 months postinjury in patients with ATSCI.

### Specific objectives

1. Determine the BASIC score of patients with ATSCI at admission.
2. Determine the AIS of patients with ATSCI at admission and 3 months postinjury.
3. Classify patients with acute SCI for neurological injury severity using the BASIC score.
4. To correlate BASIC score with AIS at admission and 3 months postinjury.

### Scope of the study

This was a prospective, longitudinal study carried out in the Neurosurgery Unit, Department of Surgery, at the National Hospital, Abuja over 1 year. It determined the AIS grade and BASIC score of all the patients with acute SCI who presented to the National Trauma Centre of National Hospital, Abuja, and who met the criteria for enrolment into the study. The study also compared the ISNCSCI and BASIC score with respect to validity of neurological injury classification (AIS score) at presentation and prediction of AIS score improvement at discharge and at 3 months after injury.

## Hypotheses

### Null hypothesis

There is no significant difference between the accuracy of the AIS and the BASIC score in classifying patients with ATSCI for neurological injury severity and AIS score improvement at 3 months postinjury.

### Alternative hypothesis

There is a significant difference between the accuracy of the AIS and the BASIC score in classifying patients with acute SCI for neurological injury severity and AIS score improvement at 3 months postinjury.

## Methods

### Study area

This study was done in the Neurosurgery Unit of the Department of Surgery, National Hospital, Abuja, located in the Abuja metropolis within the Federal Capital Territory, situated in the north central geopolitical zone of Nigeria. The Federal Capital Territory has an estimated population of about 1,405,201 from the 2006 national census figure.^24^

National Hospital, Abuja, is a tertiary health institution that offers neurosurgical care to the residents of the Federal Capital Territory and the neighboring states of Niger, Kogi, Kaduna, Nasarawa, and Benue states. The hospital has a capacity of 407 beds.

### Study design

This was a prospective, correlational, hospital-based study of all consecutive consenting patients who presented to the Neurosurgery Unit of the National Hospital, Abuja, with clinical and radiological features of ATSCI.

### Study population

All consenting patients who presented to the neurosurgery unit of National Hospital, Abuja with clinical and radiological features of ATSCI.

## Inclusion and Exclusion Criteria

### Inclusion criteria

All adult patients with acute SCI who had MRI of the injured spine within 72 h of injury.

### Exclusion criteria

1. Patients who did not give informed consent.
2. Patients below age 18.
3. Patients with preexisting clinical SCI.
4. Patients with penetrating acute SCI.
5. Patients with impaired consciousness having Glasgow Coma Scale ≤ 14.
6. Patients unable to obtain MRI imaging within 72 h of injury.
7. Patients with cauda equina injuries.
8. Patients with MR images acquired using MRI machine with field strength less than 1.5T.
9. Patients who had major emergency surgery related to their traumatic injuries before commencing neurosurgical management.

### Study period

Data collection was done over a 12-month period. The patients were followed up 3 months postinjury. Data collection ended in the 12th month, and all collected data were analyzed immediately following the end of data collection.

### Sample size

The sample size was calculated using the Cochran’s formula.^25^

- Cochran’s sample size calculation formula, *n* = $\frac{z\alpha^{2}\;\cdot \;p\;\cdot \;q}{D^{2\cdot }}$

Where:

*n* = minimum sample size when the population is ≥10,000

*n_f_* = minimum sample size when the population is <10,000

*N* = estimate of the population size (obtained from previous facility-based study)^26^

*z*α = standard normal deviation corresponding to 95% confidence level, read from Table of Statistical Figures = 1.96

*p* = expected prevalence estimated at 4.4 per 10,000 (0.044)^27^*q* = 1-P (1-0.044) = 0.956

*D* = desired degree of accuracy (precision) set at 4% (0.04).

The sample size, $\begin{array}{l} \text{n}=\frac{1.96^{2}\times 0.044\times 0.956}{{\left(0.04\right)}^{2}}\\ \text{n}=100.99=101 \end{array}$

$$\begin{array}{l} n_{f}=\frac{n}{1+\left(n/N\right)}\\ \;\;\;\;\;\;\;=101/1+\left(101/133\right)=101/1.76=57.38 \end{array}$$

Allowing for a 10% attrition rate, *n* = 63.

The sampling technique was consecutive sampling.

### Research team

Recruitment of the patients was done by the lead author who also carried out patient clinical assessment and evaluation of MR images (in conjunction with the blinded neuroradiologist) and participated in neurosurgical management, assisted by other senior registrars in the Neurosurgery Unit during the study period. A consultant radiologist performed image analysis and reporting.

### Research tools

- A specifically designed study proforma for obtaining patient information.
- The AIS form to document neurological examination findings.
- A piece of cotton wool and a new 24-gauge needle for neurological examination.
- A patellar reflex hammer.
- RadiAnt Digital Imaging and Communications in Medicine (DICOM) viewer software 4.0.3 (64-bit) for viewing MR images.
- Philips Multiva 1.5T MRI machine.

### Study protocol

1. The patient arrived at the National Hospital Trauma Center.
2. The patient was fully resuscitated and spine MRI was done within 72 h of injury.
3. Patient enrollment was done.
4. The patient was managed appropriately for clinical condition in line with unit protocol.
5. The patient’s post-resuscitation neurological assessment according to the ISNCSCI was carried out by the researcher or any other neurosurgery senior registrar on-call and AIS was determined.
6. Findings were recorded in the study proforma.
7. Using preexisting unit protocol, the patient acquired spine MRI (including T2 axial) within 72 h post-admission except if contraindicated. MRI scans were performed on a 1.5T Philips Multiva machine (Philips N.V.). Sequences followed institutional protocol for SCI as follows: sagittal T1W and T2W fast spin echo (FSE) and axial T2W FSE. Axial T2W FSE imaging was performed with the following parameter: time to repetition (TR) 3000 ± time to echo (TE) 90 ± slice thickness 3 mm. Sagittal T2W FSE imaging had these parameters: TR 3000 ± TE 90 ± slice thickness 3 mm. Acquisition matrix was 256 × 256, and field of view was 149–24.8 mm.
8. Acquired MR images were analyzed under the supervision of a consultant radiologist with interest in neuroimaging. DICOM viewer installed on a high-definition laptop/desktop computer was used.
9. For each patient, two side-by-side image windows were opened on the DICOM viewer.
10. The T2 sagittal noncontrasted image was placed into the first window for viewing.
11. A rostral cut of the sagittal view was taken at a point well away from obvious gross spinal column abnormality or spinal cord signal abnormality.
12. If a rostral cut was not possible, a caudal cut at a point below any gross spinal column abnormality was taken.
13. The axial T2 view automatically fills the second window.
14. The sagittal T2 image was then scanned rostrocaudally or caudorostrally for the single location with the worst-appearing traumatic lesion.
15. The transverse cut indicator was placed at that identified level. That level is the single injury epicenter for obtaining the axial cut. The corresponding axial view at that point automatically displayed in the second window.
16. Spinal cord T2 signal intensity pattern was observed.
17. If any was found, it was described. It was then COMPARED VISUALLY with any of those described in the BASIC score pictorially. If it matched any of those described in the BASIC score, it was assigned the appropriate score viz BASIC score 0, 1, 2, 3, or 4. The best match was chosen. Where no match was possible, this was recorded as such.
18. The BASIC score was recorded in the patient’s study proforma.
19. The patient was managed operatively or nonoperatively following unit protocol.
20. The patient’s AIS was re-evaluated again exactly 3 months postinjury.
21. These steps were repeated in sequence for all enrolled patients.

Lesions that indicated the single injury epicenter were as follows:

- Spinal cord T2 hypo-intensity area. This suggests intramedullary hematoma.
- In the absence of the above, it would be intramedullary T2 hyperintensity only. A cut was made by identifying the rostral extent and the caudal extent and finding the middle point or the point with greatest extent of intramedullary signal change. This was the single injury epicenter for obtaining the axial cut.
- In the absence of both of previous instances, a point of maximal obvious spinal cord compression was identified, and a cut was taken at its midpoint. This point was the single injury epicenter for obtaining the axial view.
- If all of the aforementioned were not present, then the point of maximal canal narrowing evidently because of loss of spine alignment was identified. This point was the single injury epicenter for obtaining the axial cut.
- If none of the aforementioned was found, then the point of obvious loss of spinal alignment was identified. Then the point of maximal encroachment upon the spinal canal was identified and was the single injury epicenter from which the axial cut was obtained.
- If none of the aforementioned was identified, then the point of either bony or soft tissue disruption with maximal encroachment on the spinal canal was identified as the single injury epicenter from which the axial cut was obtained.

At exactly 72 h post injury, the patient underwent another clinical neurological examination based on ISNCSCI. The motor scores and AIS grade at this point were the final and documented admission motor score and AIS grade. These were recorded in the patient’s study proforma.

The patient was followed up post-discharge, and the motor score and AIS at 3 months were documented.

At the end of the study period, all data obtained were analyzed and recorded in tables, charts, and graphs.

The correlation of BASIC score with AIS was done for each patient.

## Ethical and Other Considerations

Ethical clearance was sought and obtained from the Research and Ethical Committee (Institutional Review Board [IRB]) of National Hospital, Abuja (NHA/EC/100/2019). Informed consent was also obtained from all patients who met the criteria for inclusion in the study.

### Data collection method

1. IRB approval was obtained from the Ethical Review Committee of the National Hospital, Abuja.
2. All the consultants and resident doctors in the Division of Neurosurgery and clinic nurses were informed of the study.
3. A consultant radiologist was involved in evaluation of the MRI.
4. The objectives of the study were explained in detail to the patients/relatives, and consent was duly obtained before data collection.
5. A full history and physical examination were performed to identify patients to be included in the study.
6. Appropriate MRI was done for all the patients enrolled into the study as indicated in the standard guideline for the management of the patient.
7. Review of MRI was done in collaboration with the consultant radiologist.

## Data Analysis/Statistical Methods

Data collected were analyzed using SPSS(R) version 23 statistical software. The results were presented in forms of tables, pie charts, and graphs. Quantitative variables were expressed as mean and standard deviation. The analysis of the variable was done using Chi-square test or Fisher’s exact test for categorical variables and Spearman’s rank correlation test for correlation analysis. A two-tailed *p*-value of <0.05 was used as the level of significance.

Statistical correlation between individual BASIC scores at admission and the admission and 3-month postinjury AIS were done.

## Results

### Sociodemographic characteristics and patient presentation

A total of 65 patients were enrolled into the study. The demographic distribution of study participants is seen in Table 1. Thirty-eight patients (58.5%) belonged to the manually skilled occupational group,^28^ whereas 6 (9.2%) were top professionals including public and civil servants, 9 (13.8%) were unskilled workers, and 12 (18.5%) were students. The pattern of patient presentation is also shown in Table 1.

**Table 1.** Characteristics of Study Participants

Variable	Frequency (*n* = 65)	Percent
Age (years)		
Mean	39.1 ± 12.3	
Median	39.0	
<20	3	4.6
20–29	15	23.1
30–39	15	23.1
40–49	22	33.8
≥50	10	15.4
Sex		
Male	53	81.5
Female	12	18.5
Time since accident/trauma (hours)		
Mean	59.5 ± 16.5	
Median	72.0	
<12	11	16.9
12–23	20	30.8
24–35	12	18.5
36–47	4	6.2
≥48	18	27.7
Mechanism of trauma		
RTC	57	87.7
Fall	6	9.2
Pedestrian victim	2	3.1
AIS at admission		
A	47	72.3
B	8	12.3
C	4	6.2
D	4	6.2
E	2	3.1
Time to MRI		
Within 24 h	7	10.8
Beyond 24 h but within 48 h	20	30.8
Beyond 48 h but within 72 h	72	58.5

AIS, American Spinal Injury Association Impairment Scale; MRI, magnetic resonance imaging; RTC, road traffic crash.

The results of neurological examination carried out 72 h post-injury (at admission) are shown in Table 1. Complete SCI (AIS grade A) was 47 (72.3%), whereas 2 (3.1%) were AIS grade E. Five patients (7.7%) had thoracic spine injury, whereas 60 (92.3%) had cervical spine injury.

**FIG. 1.**
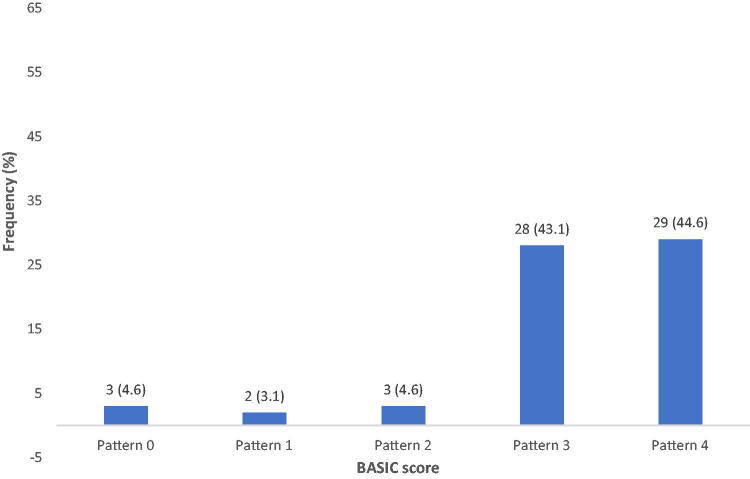
BASIC scores. BASIC, Brain and Spinal Injury Center.

BASIC scores are shown in Figure 1. BASIC 4 was the most frequent (44.6%), whereas BASIC score 1 (3.1%) was the least frequent.

The association between the BASIC score and the AIS grade at admission is shown in Table 2. This was statistically significant (Fisher’s exact test, *p* < 0.001).

**Table 2.** Association Between BASIC Score and AIS Grade at Admission

BASIC score	AIS grade at admission					
	A	B	C	D	E	Total
Pattern 0	0 (0)	0 (0)	0 (0)	2 (66.7)	1 (33.3)	3
Pattern 1	0 (0)	0 (0)	0 (0)	1 (50.0)	1 (50.0)	2
Pattern 2	0 (0)	1 (33.3)	2 (66.7)	0 (0)	0 (0)	3
Pattern 3	21 (75.0)	5 (17.9)	2 (7.1)	0 (0)	0 (0)	28
Pattern 4	26 (89.7)	2 (6.9)	0 (0)	1 (3.4)	0 (0)	29

χ^2^ = 81.527; df = 16; *p* < 0.001; Fisher’s exact test, **p < 0.001.

AIS, American Spinal Injury Association Impairment Scale; BASIC, Brain and Spinal Injury Center.

The injuries involved different spinal levels, and the corresponding maximum canal compromise and maximum spinal cord impingement were identified for each patient. Injury at C5 disc level was most frequent (35.4%), whereas T11 disc and T12 body were jointly least frequent at 1.5% each. The level of maximum cord impingement was most commonly seen at C5 body level (27.7%) and least at T11 disc and T12 body, respectively (1.5%).

Three patients (7.7%) had spine traction using Gardner–Wells tongs, whereas 62 (95.4%) did not undergo spine traction. Surgical decompression ± spine fixation at the injured spinal levels were done for 27 (41.5%) of the patients, whereas 38 (58.5%) did not have any operative decompression.

Among the patients who had surgical decompression, 15 (55.6%) had anterior cervical discectomy and fusion, 5 (18.5%) had anterior cervical corpectomy and fusion, whereas 5 (25.9%) had thoracic spine laminectomies ± pedicle screw instrumentation.

The majority of patients stayed on admission for 45–59 days (35.4%), whereas (26.2%) stayed 60 days or more. The mean length of hospital stay was 46.9 ± 17.9 days, whereas the median length of stay was 50 days. Physical rehabilitation therapy commenced while on admission and continued on outpatient basis after discharge.

**FIG. 2.**
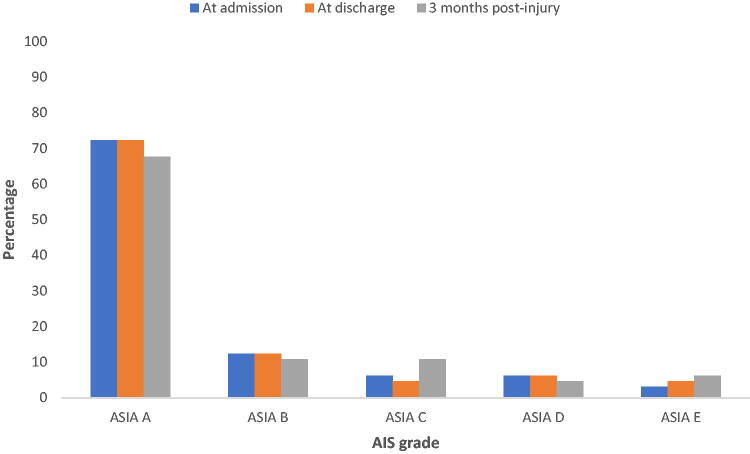
AIS grade at admission, discharge, and 3 months postinjury. AIS, American Spinal Injury Association Impairment Scale.

After the patients were discharged, they were followed up at the surgical outpatient department of the hospital where their neurological examination according to the ISNCSCI was carried out and AIS grade was determined at the third month postinjury. AIS grade A was most frequent (67.7%), whereas AIS grade D (4.6%) was least frequent. These are all shown in the Table 3 and Figure 2. The association between the AIS grade at 3 months postinjury and the BASIC score is also shown in Table 3.

**Table 3.** Association Between BASIC Score and AIS Grade at 3 Months Postinjury

BASIC score	AIS grade at 3 months post-injury					
	A (*n* = 44)	B (*n* = 7)	C (*n* = 7)	D (*n* = 7)	E (*n* = 4)	Total (*n* = 65)
Pattern 0	0 (0)	0 (0)	0 (0)	0 (0)	3 (100)	3
Pattern 1	0 (0)	0 (0)	0 (0)	1 (50.0)	1 (50.0)	2
Pattern 2	0 (0)	0 (0)	2 (66.7)	1 (33.3)	0 (0)	3
Pattern 3	19 (67.9)	4 (14.3)	5 (17.9)	0 (0)	0 (0)	28
Pattern 4	25 (86.2)	3 (10.3)	0 (0)	1 (3.4)	0 (0)	29

AIS, American Spinal Injury Association Impairment Scale; BASIC, Brain and Spinal Injury Center.

The AIS grade of 55 (84.6%) of the participants did not change between admission and 3 months postinjury (Kappa’s measure of agreement = 0.6). The AIS score improved in the remaining 10 (15.4%), disaggregated into improvement from A to B, B to C, C to D, and D to E in 3 (4.6%), 4 (6.2%), 1 (1.5%), and 2 (3.1%) of the participants, respectively (McNemar–Bowker Chi-square = 10.000; *p* = 0.040).

Among the patients who experienced AIS grade improvement, the greatest change occurred from AIS grade B to C (6.2%), followed by change from AIS grade A to B (4.6%). Taken together, the greatest AIS grade change occurred at the injury spectrum deemed by the ISNCSCI to be most severe, that is, AIS grades A and B (total percentage change 10.8%).

Further analysis was done to determine the factors in this study that were associated with improvement in AIS grade at third month postinjury.

It was found that patients in whom the bulbospongiosus reflex was absent at 72 h postinjury were less likely to experience an AIS grade improvement at 3 months postinjury, whereas those patients who had the reflex present at 72 h postinjury were more likely to experience AIS grade improvement at 3 months postinjury (statistically significant at Fisher’s exact test, *p* = 0.025).

Patients with the lower BASIC scores of 0–2 were more likely to experience an AIS grade improvement at the third month postinjury, whereas the patients with higher BASIC scores of 3–4 were less likely to experience the AIS grade improvement. This association with BASIC score was statistically significant at Fisher’s exact test, *p* = 0.006.

In addition, both spinal traction and surgical decompression were found to have statistically significant association with AIS grade improvement: patients who had spinal traction had greater likelihood of AIS grade improvement (Fisher’s exact test, *p* = 0.579), and patients who underwent surgical decompression were more likely to experience AIS grade improvement at 3 months postinjury (Fisher exact test, *p* = 0.079).

Finally, the patients who had surgical decompression early (<6 days postinjury) were more likely to experience AIS grade improvement at 3 months postinjury (significant at Fisher’s exact test, *p* = 0.196).

### Multiple regression analysis of the factors associated with AIS grade improvement at 3 months postinjury

The majority of the patients (38, 58.5%) who participated in the study did not have surgery for decompression and/or spine fixation even when they otherwise required surgery (as determined by the managing neurosurgeon), whereas only 27 (41.5%) had surgery as required. The potential effect of surgery whose overall aim is to improve outcome, confounding the AIS grade improvement at 3 months postinjury, was accounted for in a multiple logistic regression analysis.

The result shows that the patients who had surgery were 32 times more likely to experience AIS grade improvement than those who did not. However, majority of our patients did not have surgery even when they required it.

In addition, the likelihood of AIS grade improvement increased 1.79 times in those patients who had return of bulbospongiosus reflex by 72 h postinjury compared with those for whom there was no return.

Finally, the chances of AIS grade improvement reduced by 4.72 times as the BASIC score increased from 0 to 4.

### BASIC score association with AIS grade at admission

The lower BASIC scores of 0 and 1 were found only in patients who had AIS grades D and E. One (33.3%) out of the 3 patients with BASIC score 2 had AIS grade B, whereas the other 2 (66.7%) were grade C. BASIC score 2 was not found among the patients who had AIS A, nor among those with AIS grades D or E.

No patient who presented with AIS grade A had a BASIC score of 0–2. Rather, all the patients presenting with severe neurological impairment (AIS grade A) had BASIC scores of 3 and 4. BASIC score 4 was found mostly among patients with AIS grades A (89.7%) and AIS grade B (6.9%), total 96.6%, but 1 patient with BASIC score 4 (3.4%) was found to have AIS grade D.

These findings were statistically significant (Fisher’s exact test; *p* < 0.001).

### The BASIC score association with the AIS grade at 3 months postinjury

When the analysis was done for association between the BASIC score and the AIS at 3 months postinjury (Table 3), it was found that the lower BASIC scores of 0 and 1 were found only among the patients who had AIS grades D and E. No patient with BASIC scores 0 or 1 was found with AIS A or B. BASIC scores 0 and 1 therefore appear to represent the mild end of SCI severity spectrum.

BASIC score 2 was found only among patients with AIS C (2, 66.7%) and AIS D (1, 33.3%). Of the patients with BASIC score 2, none was found to have AIS grades A or B, and none had grade E. Therefore, BASIC score 2 appears to represent the intermediate portion of the classification of SCI severity using BASIC score.

BASIC score 3 was found predominantly among the patients who had AIS grades A and B (82.2%), though few (17.9%) were found among patients with AIS grade C. This appears to be an overlap with patients who had BASIC score 2 and AIS grade C. This suggests that although the BASIC score 3 tends toward the severe end of the injury severity spectrum, a few patients with the score may actually have a high likelihood of AIS grade improvement. This increases the possibility that the most severe end of the spectrum is actually represented by BASIC score 4.

Among the patients who had BASIC score 4, all had AIS grade A or B: 86.2% were grade A, whereas 10.3% were grade B. Only one patient with BASIC score 4 was found with AIS grade D (3.4%).

The association of BASIC score with AIS at 3 months postinjury is statistically significant (Fisher’s exact test; *p* < 0.001).

### BASIC score prediction of patients with AIS grade A at admission who are likely to improve from those less likely to improve

Among the patients who had BASIC score 4, there were 29, of which 89.7% had AIS grade A at admission, and by the third month postinjury, 86.2% remained at AIS grade A, a change of 3.5%. For patients with BASIC score 3, there were 28, of which 75% were at AIS grade A at admission, but by the third month postinjury, 67.9% remained at AIS grade A, a change of 7.1%. Therefore, for patients with AIS grade A, most patients had BASIC score 4, whereas those with the same AIS grade with BASIC score 3 tend to improve. For each grade of AIS, the better the BASIC score (0 and 1), the higher the likelihood for improvement. This association is statistically significant (Fisher’s exact test; *p* < 0.001).

### Correlational analysis of BASIC score and AIS grades

When the strength of correlation between the BASIC score and AIS grades obtained at admission and at 3 months postinjury were analyzed, there is moderate correlation between the BASIC score and the AIS grade at admission (Spearman correlation coefficient; *p* = 0.586) and between the BASIC score and the AIS grade at 3 months postinjury (Spearman correlation coefficient; *p* = 0.589), especially in patients with cervical SCI.

For thoracic SCI, no result was obtainable by analysis; however, when all the patients were taken as a whole, the value of *p* dropped a little but strength of correlation remained moderate at *p* = 0.532 for admission AIS grade and *p* = 0.546 for AIS grade at 3 months postinjury (Table 4).

**Table 4.** Correlation Matrix of BASIC Score and ASIA Grades

Spearman correlation coefficient (*p*)				
Variable	BASIC score	AIS at admission	AIS at discharge	AIS 3 months after
Cervical level (*n* = 60)				
BASIC score	1	—	—	—
AIS at admission	0.586	1	—	—
AIS at discharge	0.587	0.999	1	
AIS 3 months after	0.589	0.931	0.932	1
Thoracic level (*n* = 5)				
BASIC score	—	—	—	—
AIS at admission	—	1	—	—
AIS at discharge	—	1	1	
AIS 3 months after	—	1	1	1
Overall (*n* = 65)				
BASIC score	1	—	—	—
AIS at admission	0.532	1	—	—
AIS at discharge	0.536	0.999	1	—
AIS 3 months after	0.546	0.936	0.936	1

AIS, American Spinal Injury Association Impairment Scale; ASIA, American Spinal Injury Association; BASIC, Brain and Spinal Injury Center.

The sensitivity of the BASIC score for severe injury is 55.3%, whereas its specificity for severe injury is 83.3% (Table 5).

**Table 5.** Characteristics (Sensitivity, Specificity, PPV, and NPV) of BASIC Score Using AIS at Admission as Gold Standard

BASIC score	AIS at admission		
	A	≥B	Total
Sensitivity and Specificity			
Pattern 4	26 (55.3%)—Sensitivity	3	29
≤Pattern 3	21	15 (83.2%)—Specificity	36
Total	47	18	65
PPV and NPV			
Pattern 4	26 (89.7%)—PPV	3	29
≤Pattern 3	21	15 (41.7%)—NPV	36
Total	47	18	65

AIS, American Spinal Injury Association Impairment Scale; BASIC, Brain and Spinal Injury Center; NPV, negative predictive value; PPV, positive predictive value.

The BASIC score also has a positive predictive value of 89.7% for predicting severe SCI AIS A (Table 5).

**FIG. 3.**
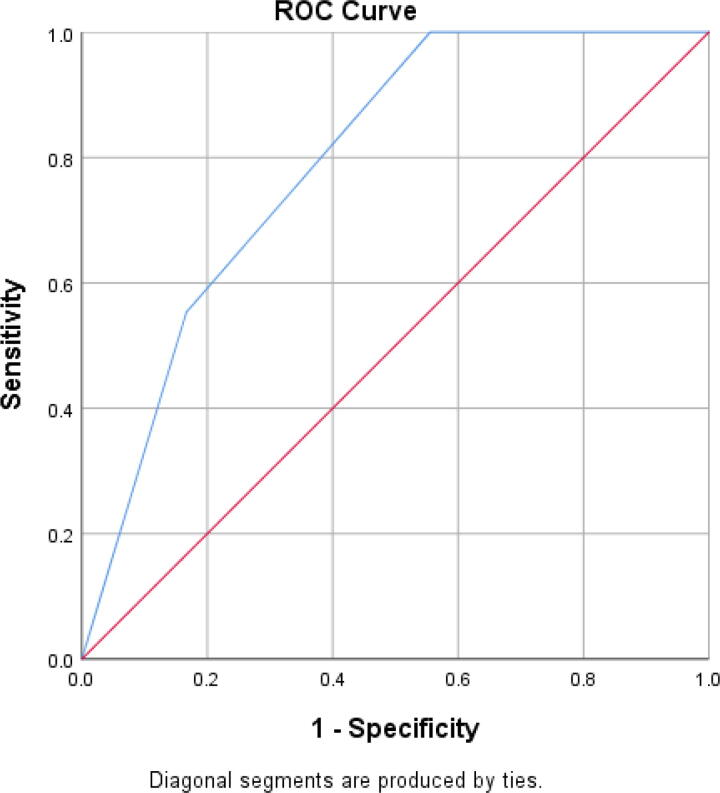
ROC curve of BASIC score using AIS at admission as gold standard. AIS, American Spinal Injury Association Impairment Scale; BASIC, Brain and Spinal Injury Center; ROC, receiver operating characteristics.

Receiver operating characteristics (ROC) curve is shown in Figure 3. Area under curve = 0.793 (79.3%); highest likelihood ratio = 3.311 with sensitivity of 0.553; and 1-specificity of 0.167 correspond to a BASIC score above 3. This shows that the best discriminant value of BASIC score is a score above 3, that is, 4.

## Discussion

### Sociodemographics and patient presentation

The majority of the patients were aged between 19 and 49 years (55, 84.6%), with age range of 19–67 and mean age 39.1 ± 12.3 years. This is similar to an earlier study from our facility^26^ that recorded mean age of 37.0 ± 13.6. It however differed from the study that derived the BASIC score, in that the age range was 18–94 years and mean age was 56 years.^9^ This difference may be partly explained by the relatively young age of the Nigerian population compared with the fact that the setting of the original work is a community of older, more affluent individuals with longer life expectancy.

The sex distribution also shows that males were affected more than females with male-to-female ratio of 4.4:1, similar to other studies in which the male-to-female ratios were 2.3:1 and 4.2:1, respectively.^9,26^

The largest occupational class of patients was the manually skilled group^28^ (58.5%), followed by students (18.5%). These are the set of people who embark on frequent interstate travels in our environment and are exposed to hazards of poor vehicular and road infrastructure.

The predominant injury mechanism was by far road traffic crash in which the victims were vehicle occupants (87.7%). Pedestrian victims of road traffic accidents accounted for 3.1%, which when added to the above totals 90.8% from road traffic accidents. Taken together with the commonest occupational groups affected, the implication is that this is so because these are the individuals who are more likely to engage in road travel in the course of daily work thus being exposed to heightened risk from poor roads and ill-maintained vehicles. This is, however, in contrast to the study from which BASIC score was derived, which showed that the leading mechanism of injury was falls/jump (53%) versus motor vehicle collision, bicycle accident, and pedestrian road traffic accidents totaling just 32%. Here again, the sociodemographic characteristics and the different physical environment of a developed country (USA) with aging population and superb road transport infrastructure explain these marked differences.

Most of the patients at our center presented between 12 and 23 h postinjury (30.8%). The mean period elapsed from injury to presentation was 59.5 ± 16.5 h. To the best of our knowledge, there is no local study that determined time elapsed between injury and admission. The closest to that is an earlier study from our center that determined mean time from injury to acquisition of MRI scan. In that study,^29^ the mean time to MRI scan was 69.1 ± 59.0 h, which is consistent with our study in which all our patients had MRI within 72 h of injury. This may have implications for prognosis because attempts to save or rescue injured neural tissue are always a race against time, and a lot of hours are already being expended before spinal cord decompression is availed for the patient.

From the admission neurological examination (done at 72 h postinjury) to determine severity of neurological impairment, the majority of patients (72.3%) had complete SCI (AIS grade A). The remaining patients (27.7%) were distributed from grade B to E, with E being the fewest (3.1%). This is similar to a previous work at our facility^26^ that recorded AIS grade A as the most common (42.5%). This is, however, different from the study from which BASIC score was derived^9^ in which AIS grade A contributed only 28%, whereas AIS grade D was the commonest at 30%.

Also, in their systematic review, Vafar Rahimi et al.^30^ found that in developing countries, complete injury is commoner than incomplete (56.5% vs. 43%). These confirm that complete SCI with severe neurological impairment AIS A is indeed more prevalent in our third-world environment. From our study, and also the original study, penetrating SCI and SCI with overt transection were excluded, and these are some of the worst injury morphologies/mechanisms for which patients and relations seek care and answers concerning prognosis. Therefore, in traumatic SCIs in which spinal cord gross morphology is not preserved, the BASIC score may not be applicable.

Sixty patients (92.3%) had injury at cervical spinal cord level, whereas 5 (7.7%) had injury at thoracic spine level. This is similar to finding of a previous study at the same center,^29^ whereas elsewhere in Nigeria, Williams Yongu et al.^31^ also found that cervical spine was more commonly injured (53.3%), whereas Vafa Rahimi^30^ had earlier reported that thoracic spinal cord is the most commonly injured segment in developing countries.

The commonest spine segment injured was C5 disc level (35.4%). In other studies, more injuries were recorded at C6/C7 level.^31,32^ Also, it was at C5 disc level that maximum cord impingement most frequently occurred (33.8%).

The distribution of the BASIC score followed the ASIA impairment grade distribution, where BASIC 4 signifying highest injury severity was the most frequent (29, 44.6%).

This finding is at variance with BASIC score distribution seen from the study from which BASIC score was initially derived, because BASIC score 2 predominated (30%), whereas BASIC score 4 was the least (8%). This may be explained by the fact of the disparity between the two studies with respect to injury mechanism and severity of injury and neurological impairment at presentation. The BASIC scores are therefore consistent with the characteristics of the local study population.

## Patient Management and Treatment

The majority of our patients (38, 58.5%) did not undergo decompressive surgery or fixation. This was irrespective of the necessity for surgery as determined by the managing neurosurgeon. Such nonoperative management was constrained by patient poverty. Williams Yongu et al.^31^ reported that all their patients over 3 years were managed nonoperatively even though no reason was given for this.

Multiple logistic regression analysis revealed that surgery is a factor in AIS grade improvement and that patients who had surgery had 32 times greater likelihood of AIS grade improvement at 3 months postinjury, and this was without any particular prejudice to the timing of the surgery.

### Correlation of BASIC score with AIS grade at admission and 3 months post-injury

BASIC score showed moderate correlation with the AIS grade at admission (Spearman correlation coefficient, *p* = 0.532). This shows that BASIC score can be used as a surrogate to AIS in the neurological evaluation of patients with ATSCI at presentation.

At 3 months postinjury, the BASIC score has a correlation with AIS of 0.546, similar to but slightly better than correlation with admission AIS grade. This shows that BASIC score can predict the prognosis of ATSCI over time. The strength of correlation is lower than obtained in the work that derived BASIC score (Pearson correlation coefficient 0.88).^9^ However, the reason(s) for this difference could be that the authors in that study used AIS grade at discharge, which likely was obtained earlier in time than our corresponding later AIS grade check, which was 3 months postinjury. Mean admission duration was 23 ± 24 days in that study. Therefore, our study allowed more time for AIS grade improvement to manifest compared with the original study where admission and discharge AIS gradings were closer in time.

Another potential reason could be that the original study was based exclusively on cervical SCI, whereas our study included thoracic spinal cord because we believe that similar pathoanatomic factors apply to both areas of the spinal cord. But more importantly, it is possible that the severity of SCI that preponderates in our environment, with concomitant poor mode of transportation to hospital, explains these differences.

### BASIC score, classification of injury severity, and prognostication

Our work also showed that the BASIC score performs well in identifying and predicting those SCI patients who, though initially at admission were classified as AIS grade A, went on to experience AIS grade improvement, separate from those who remained at AIS grade A up to 3 months after injury. This shows improved accuracy of prognostication of neurological recovery after injury.

The ROC analysis shows that the best score for discriminating the severity of injury using BASIC score is above 3 (3.5). However, the categories in the scoring system are represented as discrete numbers. Therefore, it is apparent that BASIC score 4 does best in discriminating injury severity, and the lower scores of 0–3 may all indicate relative mildness of injury.

This has implications for the use of BASIC score in stratifying patients for enrollment into interventions aimed at promoting neurological recovery after spinal injury. From this study it is apparent that only patients with scores of 4 represent truly severe injury and that scores below 4 represent the spectrum of comparatively milder injury.

Finally, our study found that patients not operated on had longer duration of hospital stay than those who had surgery. Also, the earlier the surgical intervention, the greater the likelihood of experiencing AIS grade improvement. This is in line with other studies such as Surgical Timing in Acute Spinal Cord Injury Study.^32^

## Conclusions

This study shows that the BASIC score can be used to classify severity of neurological injury among patients with ATSCI in Nigeria. Injuries assigned BASIC score 0–3 can generally be regarded as comparatively less severe than BASIC score 4, even when such are initially clinically classified as complete according to the AIS grade.

The patients assigned BASIC score 4 have the most severe injury indeed and are least likely to experience neurological improvement, and this fact may be useful in the counseling of patients and their relatives.

The BASIC score also helps with prognostication of recovery using durable and reproducible patho-anatomical injury features that have a predictable natural history rather than using functional impairment alone.

## Limitations of the Study

1. The 1-year study duration does not allow longer patient follow-up.
2. Patient poverty did not allow some patients to avail optimum care as evidenced by patients who otherwise required surgery for decompression and spine fusion not being able to obtain it because of a lack of funds to pay for surgery and implants.

## Recommendations

Following from the outcomes of this study, the following recommendations are offered:

1. BASIC score can be applied in the injury severity stratification of patients with ATSCI.
2. The BASIC score can be used in patient counseling concerning the possibility of AIS grade improvement and any hope of neurological recovery in patients with ATSCI. This is particularly important for patients clinically classified as having AIS grade A injuries in the absence of features of BASIC score 3 or 4, especially in resource-limited parts of the world who otherwise stand a risk of dropping down the priority list because of perceived futility of care.
3. There is need for a larger multicenter study with longer follow-up period to further evaluate and validate the BASIC score for ATSCI, especially in Low and Medium Income Country like Nigeria.

## Abbreviations Used

AIS: American Spinal Injury Association Impairment Scale/Abbreviated Injury Score
ASIA: American Spinal Injury Association
ATSCI: acute traumatic spinal cord injury
BASIC: Brain and Spinal Injury Center
DICOM: Digital Imaging and Communications in Medicine
FOV: field of view
FSE: fast spin echo
ISNCSCI: International Standards for the Neurological Classificationof Spinal Cord Injury
MRI: magnetic resonance imaging
NPV: negative predictive value
PPV: positive predictive value
ROC: receiver operating characteristics
RTC: road traffic crash
SCI: spinal cord injury
T1W: T1 weighted
T2W: T2 weighted
TE: time to echo
TR: time to repetition
